# A phase II study of vinflunine in bladder cancer patients progressing after first-line platinum-containing regimen

**DOI:** 10.1038/sj.bjc.6603118

**Published:** 2006-04-18

**Authors:** S Culine, C Theodore, M De Santis, B Bui, T Demkow, J Lorenz, F Rolland, F-M Delgado, B Longerey, N James

**Affiliations:** 1 CRLC Val d'Aurelle, Montpellier, France; 2 Institut Gustave Roussy, Villejuif, France; 3 Kaiser Franz-Josef Spital, Vienna, Austria; 4 Institut Bergonié, Bordeaux, France; 5 Centrum Onkologie Instytut, Warsaw, Poland; 6 Akademia Medyczna we Wroclaw, Wroclaw, Poland; 7 Centre René Gauducheau, St Herblain, France; 8 Institut de Recherche Pierre Fabre, Boulogne-Billancourt, France; 9 Queen Elizabeth Hospital, Edgbaston, Birmingham, UK

**Keywords:** vinflunine, phase II, transitional cell carcinoma (TCC) of the bladder

## Abstract

A multicentre phase II trial to determine the efficacy of vinflunine as second-line therapy in patients with advanced transitional cell carcinoma (TCC) of the bladder; secondary objectives were to assess duration of response, progression-free survival (PFS) and overall survival (OS), and to evaluate the toxicity associated with this treatment. Patients had tumours that failed or progressed after first-line platinum-containing regimens for advanced or metastatic disease, or had progressive disease after platinum-containing chemotherapy given with adjuvant or neoadjuvant intent. Response and adverse events were assessed according to WHO criteria and NCI-CTC (version 2), respectively. Out of 51 patients treated with 320 mg m^−2^ of vinflunine, nine patients responded to the therapy yielding an overall response rate of 18% (95% CI: 8.4–30.9%), and 67% (95%CI: 52.1–79.3%) achieved disease control (PR+SD). Of note, responses were seen in patients with relatively poor prognostic factors such as a short (<12 months) interval from prior platinum therapy (19%, including an 11% response rate in those progressing <3 months after platinum treatment), prior treatment for metastatic disease (24%), prior treatment with vinca alkaloids (14%) and visceral involvement (20%). The median duration of response was 9.1 months (95% CI: 4.2–15.0) and the median PFS was 3.0 months (95% CI: 2.4–3.8). The median OS was 6.6 months (95% CI: 4.8–7.6). The main haematological toxicity was grade 3–4 neutropenia, observed in 67% of patients (42% of cycles). Febrile neutropenia was observed in five patients (10%) and among them two were fatal. Constipation was frequently observed (but was manageable and noncumulative) and was grade 3–4 in only 8% of patients. The incidence of grade 3 nausea and vomiting was very low (4 and 6% of patients, respectively). Neither grade 3–4 sensory neuropathy nor severe venous irritation was observed. Moreover, and of importance in this particular study population, no grade 3–4 renal function impairment was observed. Vinflunine is an active agent for the treatment of platinum-pretreated bladder cancer, and these results warrant further investigation in phase III trials, either as monotherapy or in combination with other agents as treatment of advanced/metastatic TCC of the bladder.

Before the development of effective chemotherapy, median survival of patients with metastatic bladder cancer rarely exceeded 4 months. Treatment with cisplatin, approved for the treatment of advanced disease in the early 1980s, doubled median survival to 8 months (Loehrer *et al*, 1992).

The combination regimen including methotrexate, vinblastine, doxorubicin and cisplatin (M-VAC) was devised at Memorial Sloan-Kettering in the mid 1980s (Sternberg *et al*, 1988) and improved overall survival to a median slightly in excess of 12 months and became the standard of care worldwide for advanced bladder cancer (Culine, 2002).

Gemcitabine was developed in the late 1990s and, as a single agent, produced objective response rates ranging from 11 to 29% in second-line (Lorusso *et al*, 1988; Gebbia *et al*, 1999; Albers *et al*, 2002), and of 24–45% in first-line therapy (Moore *et al*, 1997; Stadler *et al*, 1997; Castagneto *et al*, 2004). A combination regimen of cisplatin and gemcitabine was developed and compared with M-VAC, producing comparable efficacy results, and a reduction of side effects (most notably severe mucositis and infections, as well as alopecia) (Von Der Maase *et al*, 2000). Based on these results, gemcitabine (in combination with cisplatin) received regulatory approval for the first-line treatment of advanced bladder cancer in the European Union as well as in several other countries. Although not approved in the US, the gemcitabine/cisplatin regimen has been widely adopted for this indication.

Recent years have witnessed the establishment of neoadjuvant chemotherapy as a treatment modality superior in survival to surgery or radiotherapy alone for muscle-invasive bladder cancer, and this observation is currently supported by large randomised trials as well as by meta-analyses (Meta-Analysis, 2003). Owing to this, and not dissimilarly from other tumours, the most active agents and combinations are currently employed in early stages of the disease.

For patients whose disease relapses or progression after initial treatment limited options exist and new active drugs are needed.

Vinflunine ditartrate (Javlor®, Pierre Fabre Médicament, Boulogne-Billancourt – France) is a novel antitubulin agent obtained by a semisynthetic process from a vinca alkaloid base showing higher antitumour activity compared with parent compounds.

Vinflunine showed definite (high or moderate) antitumour activity in 64% (seven out of 11) of xenografts tested, compared with vinorelbine which showed only moderate activity in 27% (three out of 11) (Kruczynski *et al*, 1998a, 1998b; Hill *et al*, 1999). Furthermore, a preclinical study effects of vinflunine on a murine bladder cancer cell line (MB-49) showed clear cytotoxic activity (Bonfil *et al*, 2002). Vinflunine was thus considered a good candidate for further clinical study in bladder cancer.

Single agent vinflunine was studied in several clinical phase I trials utilising different schedules (Delord *et al*, 2001; Bennouna *et al*, 2003; Johnson *et al*, 2005), leading to the selection of the 3 weekly schedule for phase II evaluation.

## PATIENTS AND METHODS

### Objectives

This study was an open-label, multicentre, noncomparative phase II trial designed to determine the efficacy of vinflunine in patients with advanced transitional cell carcinoma (TCC) of the bladder previously treated with one platinum-based line of chemotherapy. The primary objective was to assess efficacy in terms of tumour response rate; secondary objectives were to assess duration of response, progression-free survival (PFS) and overall survival (OS), and to evaluate the treatment related toxicity.

The protocol and its amendments were submitted to Independent Ethics Committees according to local requirements. The study was conducted in accordance with the ethical principles set forth in the Declaration of Helsinki and in compliance with Good Clinical and Laboratory Practices. Written informed consent was obtained before entry into the study.

### Patient selection

A total sample size of 50 evaluable patients was calculated using the 1-sample multiple testing procedure of Fleming for phase II clinical trials (Fleming, 1982). A small excess in recruitment was allowed to replace possible dropouts.

Patients were recruited from 16 European centers between November 2000 and September 2002. Eligible patients had histologically proven TCC of the bladder, which had failed or progressed after first-line platinum-containing regimens for advanced or metastatic disease, or treatment given with adjuvant or neoadjuvant intent. Previous systemic chemotherapy or radiotherapy had to have been stopped 30 days before the administration of the study drug with full recovery from all severe side effects. The presence of at least one bidimensionally measurable lesion, not previously irradiated, assessed by CT-scan or MRI performed <28 days before first day of study drug administration was required. Patients were required to be aged ⩾18 years with Karnofsky performance status (KPS) ⩾80 and an estimated life expectancy ⩾12 weeks with adequate haematological function (absolute neutrophil count ⩾2.0 × 10^9^ l^−1^, platelets ⩾100 × 10^9^ l^−1^), hepatic function (bilirubin ⩽1.5 × upper normal limit (UNL), transaminases ⩽2.5 × UNL, unless due to liver involvement), renal function (calculated clearance of creatinine ⩾40 ml min^−1^, Cockcroft-Gault formula) and a normal electrocardiogram (ECG).

### Treatment schedule

Patients were treated at the beginning of study with intravenous (i.v.) vinflunine 350 mg m^−2^ as a 10 min infusion 3 weekly based on the previous phase I trial (Bennouna *et al*, 2003). A preliminary safety evaluation, performed programwide across all ongoing vinflunine phase II trials, led to a dose reduction to 320 mg m^−2^ 3 weekly (the next lower dose level of the preceding phase I trial).

Tolerance was assessed throughout the treatment period and before each administration according to the NCI Common Toxicity Criteria (Version 2.0). All patients who received at least one cycle were considered evaluable for safety. The use of haematopoietic growth factors (G-CSF) was allowed for patients with febrile neutropenia or neutropenic infections.

Vinflunine administration was delayed by 1 or 2 weeks in the case of significant haematological or nonhaematological toxicity (grade >2 toxicity impacting a major organ except for alopecia).

If febrile neutropenia and/or grade 4 neutropenia (<0.5 × 10^9^ l^−1^) lasting ⩾7 days was observed between two subsequent administrations of vinflunine, the dose was reduced to 280 mg m^−2^ from the next cycle on. If, after dose reduction, this toxicity was seen again, the dose was further reduced to 250 mg m^−2^. If at this dose level the same event recurred, the treatment was stopped. Blood counts were performed every 2 days until recovery of ANC ⩾1.0 × 10^9^ l^−1^. No dose re-escalation was allowed after dose reduction. In case of grade 2 mucositis and/or constipation lasting >5 days, or grade ⩾3 mucositis, and/or constipation of any duration, the vinflunine dose was reduced to 280 mg m^−2^ from the next cycle on. If, after dose reduction, one of these toxicities was seen again, the dose was reduced to 250 mg m^−2^. If at this dose the event recurred, the treatment was discontinued. Each patient received at least two cycles of treatment unless one of the following occurred: early progression, unacceptable toxicity, serious intercurrent illness, other reactions which could affect the clinical status of the patient to a significant degree requiring discontinuation of the drug, or request by the patient to withdraw consent.

After the initial two cycles, tumour response was assessed. Patients with progressive disease (PD) discontinued treatment; those with stable disease (s.d.) received two additional cycles of vinflunine, were assessed a second time and treatment was continued according to the Investigator's opinion. Complete or partial responders (CR, PR) could continue treatment either until PD, toxicity or patient preference precluded further therapy.

### Baseline and treatment evaluation

Preregistration assessments included a detailed medical history, physical examination, CT or MRI scan. All positive imaging procedures at study entry were repeated every 6 weeks. An assessment of symptoms was made at study entry and then throughout treatment. Physical examination and vital signs were assessed on day 1 of each cycle. A complete blood cell count was performed at baseline (within 7 days of drug administration), before each cycle and at days 8 and 15 of each cycle, repeated every 2 days if the ANC was <1.0 × 10^9^ l^−1^ until recovery to ANC ⩾1.0 × 10^9^ l^−1^. Transaminases, alkaline phosphatase, total bilirubin, creatinine, electrolytes and total protein were assessed at every cycle. An ECG was performed at baseline and repeated every cycle.

If a patient did not progress by the end of the study, all lesions were regularly assessed until either the patient progressed or started other anticancer therapy. Response was assessed using the WHO criteria (WHO Handbook, 1979; SWOG, 1992). In order to be confirmed, CR and PR were to be maintained for at least 4 weeks, while no other type of lesion progressed or appeared. All responses and disease stabilisations were individually reviewed and confirmed by an independent radiologist, as stipulated by the study protocol.

According to WHO criteria, the duration of response was calculated for patients with confirmed response (CR or PR) from the date of registration until the date of documented progression, death, last follow-up or last news. Any responding patient alive without progression was censored at the date of commencement of new treatment or at last contact. Progression-free survival was defined as the time elapsed from registration until progression, death, lost follow-up or last information available. Progression-free survival of patients alive without progression was censored at the date of last information. Survival was defined as the time elapsed from registration to death or lost follow-up. The statistical analysis was performed on an intent-to-treat (ITT) basis on all patients registered and treated.

## RESULTS

In total, 58 patients with advanced or metastatic TCC of the bladder were included in this study. One patient died before receiving any treatment, hence was not included in the analysis. Six patients treated initially at 350 mg m^−2^ represent too small a group to justify an extensive separate analysis, therefore results are focused on the 51 patients treated at 320 mg m^−2^, the majority of whom (84%) relapsed or progressed within 12 months of initial chemotherapy.

Demographic features are summarised in Table 1. Prior therapy included surgery, radiotherapy, or local bladder instillations of either chemotherapy or BCG and all had received systemic platinum-based chemotherapy; 22 patients (43%) received M-VAC or CMV and 25 patients (49%) gemcitabine/platinum (Table 2). Prior chemotherapy was for advanced disease in 34 patients (67%) and as adjuvant or neoadjuvant chemotherapy in 17 patients (33%) (Table 3). The median treatment-free interval between completion of initial chemotherapy and vinflunine treatment was 7.5 months (range: 0.9–94.3). All patients enrolled in the study had clear evidence of PD, 61% had two or more metastatic lesions at entry and 49% had visceral involvement.

### Efficacy evaluation

Among the 51 patients treated at 320 mg m^−2^, nine obtained a partial response (18% (95% CI: 8.4–30. 9%) and 25 achieved s.d., for an overall disease control rate (PR+s.d.) of 67% (95% CI: 52.1–79.3%). Three patients could not be evaluated for response, but are kept in the denominator for the purpose of the calculation of the response rate (Table 4).

Disease control rates appeared to correlate with the interval from prior platinum treatment, with better results in late relapsing or progressing patients. Objective response rates were eight out of 34 (24%) and one out of 17 (6%) in patients previously treated in the metastatic and neoadjuvant/adjuvant setting, respectively.

Of note, objective responses were observed in three out of 22 (14%) patients who had received prior vinca alkaloids as a component of the M-VAC or CMV regimens.

Responses were predominantly seen in patients who had previously responded to chemotherapy, although numbers are too small to exclude random variation. However, five out of 25 (20%) of patients with visceral involvement achieved an objective response and responses were seen in patients with primary chemo-resistant disease (Table 5).

Of the six additional patients who started the trial at a dose of 350 mg m^−2^, only three were evaluable and none responded.

The median duration of response was 9.1 months (95% CI: 4.2–15.0). Among the 51 patients treated at 320 mg m^−2^, the median PFS was 3.0 months (95% CI: 2.4–3.8) and the median overall survival was 6.6 months (95% CI: 4.8–7.6).

Karnofsky performance status was recorded for each patient at baseline and before each treatment cycle during the study (every 3 weeks). Of 51 (22%) patients, 11 demonstrated improvement in performance status during treatment, and 27 (53%) maintained their baseline status. Only 10 patients (20%) had a worsening during treatment.

### Safety evaluation

A total of 15 cycles were administered to six patients initially treated at 350 mg m^−2^. The most frequent adverse events at this dose included leucopenia, neutropenia and anaemia, which were observed in all six patients (100%) with one fatal febrile neutropenia.

The total number of cycles administered at 320 mg m^−2^ was 197 (median four cycles, range 1–12) with median relative dose intensity of 95.5%. Of 51 (35%) patients (14% of cycles), 18 had dose reductions with the majority from 320 mg m^−2^ to 280 mg m^−2^ affecting 14 of 146 cycles (10%). Dose reductions to the lowest planned dose (250 mg m^−2^) occurred only in six cycles (4%).

During the study, 23 of 51 (45%) patients experienced grade 3–4 leucopenia (27% of cycles), with grade 3–4 neutropenia in 34 patients (67%) (42% of cycles) (Table 6), 10 (29%) of whom had received radiotherapy in the pelvic area. The median nadir value for leucocytes and neutrophils was 2.2 × 10^9^ l^−1^ and 0.6 × 10^9^ l^−1^, respectively. The median cycle at which both leucocyte and neutrophil nadirs occurred was the second cycle of treatment (on days 7 and 12, respectively). The median duration of leucopenia and neutropenia was 7 days. Anaemia was rarely severe; only 14% of patients developed grade 3 toxicity (5% of cycles). The incidence of thrombocytopenia was also low, only three (6%) patients developed grade 3 thrombocytopenia (2% of cycles). Five patients (10%) experienced febrile neutropenia and two of them died (both had received multiple courses of vinflunine). Grade 3–4 neutropenia complicated with infection were observed in three (6%) patients (1.5% of cycles). Two (4%) patients had grade 3–4 infections without neutropenia (1% of cycles).

Nonhaematological toxicities were manageable. Only 4% of patients experienced grade 3 nausea and only 6% of patients experienced grade 3 vomiting. In all, 141 cycles (72%) were administered with prophylactic use of antiemetics according to institution protocol. Among the 28% of cycles without prophylactic antiemetics, neither severe nausea nor vomiting was observed.

Constipation occurred in 65% but was grade 3 in three patients (6%) and grade 4, in only one (2%). Among these four patients there was a strong association with previous abdominal surgery and concomitant treatment with opioids and 5HT_3_ antagonists. No cumulative effect was seen for constipation. Prophylactic laxative therapy helped to reduce the incidence of this complication. Grade 3 myalgia and fatigue were observed in only 4 and 10% of patients, respectively (1 and 3% of cycles). Grade 3–4 sensory neuropathy was not observed and the overall incidence of grade 1–2 toxicity was very low (8%) as were injection site reactions – two patients (4%) had grade 1 or 2 injection site reactions related to vinflunine.

At baseline, 10 patients (20%) had grade 1 elevation of renal function tests, which increased to grade 2 in one patient (Table 7). Despite frequent baseline involvement of the liver by disease, grade III–IV elevations of liver function tests were infrequent.

## DISCUSSION

The treatment of relapse following systemic chemotherapy for advanced or metastatic TCC is problematical. A variety of agents have been studied including ifosfamide, taxanes, oxaliplatin, pemetrexed and bortezomib and the results are summarised below.

Ifosfamide had been studied in the past, and its phase II results in previously treated patients (not all platinum pretreated) have been somewhat inconsistent with response rates of 1 and of 20% in two studies (Pronzato *et al*, 1997; Witte *et al*, 1997). Be that as it may, its use has become less frequent, due to the renal toxicity of the compound, a particular problem with bladder carcinoma patients who have received prior platinum.

A review of the recent literature concerning phase II trials in this population confirms that very limited options exist for patients who have been previously treated with gemcitabine/platinum, M-VAC or CMV combinations (Table 8). The taxanes (paclitaxel and docetaxel) have been studied in this setting, and are frequently used in clinical practice, as they have yielded objective response rates of 5–13% with response durations of 3.0–7.4 months. Severe myelosuppression (for docetaxel) and peripheral neurotoxicity (for paclitaxel) are the most common side effects. Unfortunately, when brought forward to the first-line setting, a combination of docetaxel and cisplatin was proven inferior to M-VAC (Bamias *et al*, 2004), whereas similar randomised paclitaxel/platinum combination trials were abandoned for poor accrual (Dreicer *et al*, 2004).

Recent trials with a novel platinum analogue (oxaliplatin), a dual targeted tyrosine kinase inhibitor (lapatinib) and a proteasome inhibitor (bortezomib) have yielded unimpressive results. Interesting results have been obtained with the multitargeted folate inhibitor pemetrexed, which demonstrated a high response rate of 28%, including complete responses, although of a seemingly short duration. The compound has been also studied in the first-line setting as a single agent or in combination with gemcitabine, yielding objective response rates of 33% and of 27%, respectively. Main side effects consisted of fatigue and myelosuppression. Studies have also evaluated combinations of chemotherapy and biological agents, for example, gemcitabine has been studied in combination with the farnesyl transferase inhibitor SCH66336 in the second-line treatment of patients with advanced urothelial tract cancer. Nine partial responses and one complete response were achieved in 31 assessable patients and corresponded to an overall response rate of 32.3% (95% CI: 17–51%) (Theodore *et al*, 2005). Further evaluation of combinations of this sort is clearly warranted.

The results obtained with vinflunine suggest that this compound could be among the most active agents available in this setting. The level of antitumour activity observed must be interpreted in view of the poor prognosis of the population studied, with a universal prior platinum-containing pretreatment a large proportion had had prior vinca alkaloid exposure, and the frequent presence of extensive visceral disease involvement at trial entry.

Although vinflunine exerted some severe toxicity (most importantly myelosup ion and constipation), these side effects appeared to be mostly manageable. A positive aspect of vinflunine for patients with advanced bladder cancer is its reduced potential for renal toxicity and its low level of peripheral neurotoxicity, which do not overlap with the known cisplatin side effects. Renal impairment has been reported as the most important factor preventing up to 40% of the patients with TCC of the bladder from receiving active, cisplatin-containing treatment (Dash *et al*, 2005).

In conclusion, the results of the present study demonstrate that vinflunine has clinically relevant activity with acceptable toxicity in the treatment of patients with TCC of the bladder who have failed prior platinum-containing systemic chemotherapy. Further investigation of vinflunine in advanced or metastatic TCC of urothelium tract is warranted, and a phase III trial in the second-line treatment of the disease has begun. In addition, the exploration of the role of the compound in patients with earlier stages of disease and/or renal function impairment is also of interest.

## Figures and Tables

**Table 1:** Demographic data

**No. of patients (%): 51(100)**	
*Age (years)*	
Median	63
Range	(42–81)
	
*Karnofsky performance status*	
100	12 (24)
90	16 (31)
80	22 (43)
70	1 (2)
	
Males	41 (80)
Females	10 (20)
	
*Stage at diagnosis*	
Unknown	11 (22)
0_IS_	1 (2)
I	5 (10)
II	3 (6)
III	8 (16)
IV	23 (45)
	
*Number of organs involved*	
1	20 (39)
2	19 (37)
⩾3	12 (24)
	
Lung only	7 (13.7)
Liver only	6 (11.8)
Bone only	1 (2.0)
Lymph nodes	34 (66.7)
Skin	2 (3.9)
Soft tissues	8 (15.7)
Lung+liver	9 (17.6)
Lung+bone	1 (2.0)
Liver+bone	2 (3.9)
Other organs	12 (23.5)

**Table 2:** Previous local anticancer therapies

	**Initial dose of vinflunine**	
**Treatment**	**350 mg m^−2^ ***N*** (%)**	**320 mg m^−2^ ***N*** (%)**
No. of patients	6	51
Surgery alone	5 (83.3)	31 (60.7)
Radiotherapy alone	—	2 (3.9)
Surgery+radiotherapy	1 (16.6)	15 (29.4)

**Table 3:** Prior treatment

**No. of patients (%)**	**51(100)**
Systemic platinum chemotherapy	51 (100)
For metastatic disease	34 (67)
gemcitabine/platinum	17
M-VAC/CMV	13
Other platinum regimens	4
For neoadjuvant/adjuvant	17 (33)
gemcitabine/platinum	8
M-VAC/CMV	9

**Table 4:** WHO response rate according to platinum-free interval of patients treated with 320 mg m^−2^

		**Platinum-free interval at study entry**		
	**ITT population**	**<3 months**	**3–12 months**	**⩾12 months**
No. of patients (%)	51 (100%)	19 (100%)	24 (100%)	8 (100%)
*Response rate*	9 (18%)	2 (11%)	6 (25%)	1 (13%)
	(95%CI : 8.4–30.9%)	(95%CI : 1.3–33.1%)	(95%CI : 9.8–46.7%)	(95%CI : 0.3–52.7%)
Stable disease (SD)	25 (49%)	9 (47%)	11 (46%)	5 (63%)
*Disease control*	34 (67%)	11 (58%)	17 (71%)	6 (75%)
	(95%CI : 52.1–79.3%)	(95%CI : 33.5–9.8%)	(95%CI : 48.9–87.4%)	(95%CI : 34.9–96.8%)
Progressive disease (PD)	14 (28%)	6 (32%)	6 (25%)	2 (25%)
Nonevaluable (NE)	3 (6%)	2 (11%)	1 (4%)	—

**Table 5:** Description of responders

**Age**	**Previous chemotherapy**	**Response to previous chemotherapy**	**Site of disease**	**Response to vinflunine**	**Cycles total (*N*)**	**Time to first response (months)**
62	GC	NC	Lymph nodes	PR	7	4.2
71	GC	PD	Lymph nodes+lung	PR	6	1.3
76	CMV	PR	Lymph nodes	PR	4	1.7
71	GC	PR	Lymph nodes+lung	PR	8	1.3
50	GC	PR	Liver + lung	PR	8	1.3
79	GC	PR	Liver + lung	PR	6	2.1
64	M-VAC	PR	Lymph nodes	PR	4	1.5
63	GC	NA	Lymph nodes	PR	8	1.3
45	CMV + GC	CR	Lung	PR	5	1.1

CMV=cisplatin, methotrexate, vinblastine; GC=gemcitabine, cisplatin; M-VAC=methotrexate, vinblastine, adriamycin, cisplatin.

**Table 6:** NCI CTC adverse events related to the study drug

	**No. of evaluable pts (%)**			**No. of evaluable cycles (%)**		
	**No=51**			*N*=197*		
**Adverse events**	**Overall incidence**	**Grade 3**	**Grade 4**	**Overall incidence**	**Grade 3**	**Grade 4**
Haematological*						
Anaemia	46 (90)	7 (14)	—	169 (86)	10 (5)	—
Leucopenia	43 (84)	14 (28)	9 (18)	152 (78)	41 (21)	12 (6)
Neutropenia	42 (82)	16 (31)	18 (35)	144 (74)	43 (22)	39 (20)
Thrombocytopenia	22 (43)	3 (6)	—	77 (39)	4 (2)	—
Febrile neutropenia	5 (10)	4 (8)	1 (2)	5 (3)	4 (2)	1 (1)
Infection						
Infection+G 3/4 neutropenia	3 (6)	2 (4)	1 (2)	4 (2)	2 (1)	1 (1)
Infection without neutropenia	7 (14)	—	2 (4)	10 (5)	—	2 (1)
Nonhaematological gastrointestinal						
Nausea	19 (37)	2 (4)	—	36 (18)	2 (1)	—
Vomiting	13 (226)	3 (6)	—	23 (12)	3 (2)	—
Constipation	33 (65)	3 (6)	1 (2)	63 (32)	3 (25)	1 (1)
Anorexia	4 (8)	1 (2)	—	27 (14)	—	—
Dehydration	13 (26)	—	—	4 (2)	1 (1)	—
Dysphagia/oesophagitis	2 (4)	1 (2)	—	3 (2)	1 (1)	—
Stomatitis	17 (33)	3 (6)	—	34 (17)	3 (2)	—
Flu-like symptoms						
Fatigue	34 (67)	5 (10)	—	93 (47	6 (3)	—
Pain						
Abdominal pain	19 (37)	4 (8)	—	32 (16)	6 (3)	—
Bone pain	6 (12)	2 (4)	—	9 (5)	2 (1)	—
Myalgia	9 (186)	2 (4)	—	15 (8)	2 (1)	—
Pain-other	4 (8)	—	—	5 (3)	—	—
Neurological						
Syncope	1 (2)	1 (2)	—	1 (1)	1 (1)	—
Neuropathy-sensory	4 (8)	—	—	6 (3)	—	—
Dermatological						
Alopecia	20 (39)	NA	NA	83 (42)	NA	NA
Injection site reaction	2 (4)	—	—	3 (2)	—	—
Weight loss	10 (20)	1 (2)	—	35 (18)	4 (2)	—

* One cycle was not evaluable for haematological toxicity.

**Table 7:** Renal and liver function changes on treatment

**Laboratory test**	**No. of evaluable pts**	**Overall incidence (any grade)**	**Grade 3**	**Grade 4**
Serum creatinine	50	11 (22)	—	—
Bilirubin	51	3 (6)	1 (2)	1 (2)
SGOT/AST	48	12 (25)	1 (2)	—
SGPT/ALT	49	13 (27)	1 (2)	—
Alkaline phosphatase	49	25 (51)	5 (10)	—

**Table 8:** Recent phase II trials of single agents in previously treated bladder cancer

**Drug**	**Author (ref.)**	**No. of pts**	**Evaluable pts**	**CR**	**PR**	**%**	**Duration (months)**	**TTP (months)**	**OS (months)**
Paclitaxel	Vaughn (2002)	31	31	—	3	10		2.2	7.2
	Papamichael (1997)	14	14		1	7	7.4		
	Joly (2004)	45	37	—	2	5	3.0		
Docetaxel	McCaffrey (1997)	30	30	—	4	13	4.0		9.0
Oxaliplatin	Moore (2003)	20	18	—	1	6			
Lapatinib	Wulfing (2005)	59	59	—	2	3			
Bortezomib	Sridhar (2005)	18	11	—	—	0			
Pemetrexed	Sweeney (2003)	47	47	3	10	28	3.0		9.8

## References

[bib1] Advanced Bladder Cancer Meta-Analysis Collaboration (2003) Neoadjuvant chemotherapy in invasive bladder cancer: a systemic review and meta-analysis. Cancer 361: 1927–1934 10.1016/s0140-6736(03)13580-5 12801735

[bib2] Albers P, Siener R, Hartlein M, Hartlein R, Fallahi M, Haeutle D, Perabo FG (2002) Gemcitabine monotherapy as second-line treatment in cisplatin-refractory transitional cell carcinoma – prognostic factors for response and improvement of quality of life. Onkologie 25: 47–52 11893883 10.1159/000055202

[bib3] Bamias A, Aravantinos G, Deliveliostis C, Bafaloukos D, Kalofonos C, Xiros N, Zervas A, Mitropoulos D, Samantas E, Pectasides D, Ppapakostas P, Gika D, Kourousis C, Koutras A, Papadimitriou C, Bamias C, Kosmidis P, Dimopoulos MA (2004) Docetaxel and cisplatin with granulocyte colong-stimulating factor (G-CSF) *vs* MVAC with G-CSF in advanced urothelial carcinoma: a multicenter, randomized phase III study from the Helenic Cooperative Oncology Group. J Clin Oncol 22: 220–228 14665607 10.1200/JCO.2004.02.152

[bib4] Bennouna J, Fumoleau P, Armand JP, Raymond E, Campone M, Delgado FM, Puozzo C, Marty M (2003) Phase I and pharmacokinetic study of the new vinca alkaloid vinflunine administered as a 10-min infusion every 3 weeks in patients with advanced solid tumours. Ann Oncol 14: 630–637 12649112 10.1093/annonc/mdg174

[bib5] Bonfil RD, Russo DM, Binda MM, Delgado FM, Vincenti M (2002) Higher antitumour activity of vinflunine than vinorelbine against an orthotopic murine model of transitional cell carcinoma of the bladder. Urol Oncol 7: 159–166 12474532 10.1016/s1078-1439(02)00184-9

[bib6] Castagneto B, Zai S, Marenco D, Bertetto O, Repetto L, Scaltriti L, Mencoboni M, Ferraris V, Botta M (2004) Single-agent gemcitabine in previously untreated elderly patients with advanced bladder carcinoma: response to treatment and correlation with the comprehensive geriatric assessment. Oncology 67: 27–32 15459492 10.1159/000080282

[bib7] Culine S (2002) The present and future of combination chemotherapy in bladder cancer. Semin Oncol 29(Suppl 3): 32–39 10.1053/sonc.2002.34271 12094336

[bib8] Dash A, Koppie T, Vora K, Bochner B, Galski MD (2005) The impact of renal impairment on eligibility for adjuvant cisplatin-based chemotherapy in patients with transitional cell carcinoma of the bladder. Proc Am Soc Clin Oncol 23(16S): 399s, (A4586) 10.1002/cncr.22031 16773629

[bib9] Delord JP, Stupp R, Pinel MC, Nguyen L (2001) Phase I study of vinflunine given as a 10 min intravenous infusion on a weekly schedule in patients with advanced solid tumours. Proc Am Soc Clin Oncol 20: 111a, (A441)

[bib10] Dreicer R, Manola J, Roth BJ, See WA, Kuross S, Edelman MJ, Hudes GR, Wilding G (2004) Phase III trial of methotrexate, vinblastine, doxorubicin, and cisplatin versus carboplatin and paclitaxel in patients with advanced carcinoma of the urothelium. Cancer 100: 1639–1645 15073851 10.1002/cncr.20123

[bib11] Fleming TR (1982) One-sample multiple testing procedure for phase II clinical trials. Biometrics 38: 143–151 7082756

[bib12] Gebbia V, Testa A, Borsellino N, Mauceri G, Varvara F, Tirrito ML, Sambataro D, Fallica G (1999) Single agent 2′, 2′-difluorodeoxycytidine in the treatment of metastatic urothelial carcinoma: a phase II study. Clin Ter 150: 11–15 10367539

[bib13] Hill B, Fiebig H, Waud WR, Poupon MF, Colpaert F, Kruczynski A (1999) Superior *in vivo* experimental antitumour activity of vinflunine, relative to vinorelbine, in a panel of human tumour xenografts. Eur J Cancer 3: 512–520 10.1016/s0959-8049(98)00416-x 10448309

[bib14] Johnson P, Geldart T, Fumoleau P, Pinel MC, Nguyen L, Judson I (2005) Phase I study of vinflunine given as a 10 min infusion on days 1 and 8 every 3 weeks in patients with solid malignancies. Invest New Drugs Sep 20; [Epub ahead of print] 10.1007/s10637-005-3902-0 16211365

[bib15] Joly F, Tchen N, Chevreau C, Henry-Amar M, Priou F, Chinet-Charrot P, Rolland F (2004) Clinical benefit of second line weekly paclitaxel in advanced urothelial carcinoma: a GETU phase study. Proc Am Soc Clin Oncol 23: 410, (A4619) 10.3816/CGC.2009.n.018 19692319

[bib16] Kruczynski A, Colpaert F, Tarayre JP, Mouillard P, Fahy J, Hill BT (1998a) Preclinical *in vivo* antitumour activity of vinflunine, a novel fluorinated vinca alkaloid. Cancer Chemother Pharmacol 41: 437–447 9554586 10.1007/s002800050764

[bib17] Kruczynski A, Ricome C, Astruc J (1998b) Marked antitumour activity of vinflunine, a new fluorinated vinca-alkaloid, in murine and human experimental tumours. Ann Oncol 9: 38

[bib18] Loehrer PJ, Einhorn LH, Elson PJ, Crawford ED, Kuebler P, Tannock I, Raghavan D, Stuart-Harris R, Sarosdy MF, Lowe BA (1992) A randomised comparison of cisplatin alone or in combination with methotrexate, vinblastine, and doxorubicin in patients with metastatic urothelial carcinoma: a cooperative group study. J Clin Oncol 10: 1066–1073 Erratum in J Clin Oncol 1993 February; 11:384 1607913 10.1200/JCO.1992.10.7.1066

[bib19] Lorusso V, Pollera CF, Antimi M, Luporini G, Gridelli C, Frassineti GL, Olivia C, Pacini M, De Lena M (1988) A phase II study of gemcitabine in patients with transitional cell carcinoma of the urinary tract previously treated with platinum. Eur J Cancer 34: 1208–1212 10.1016/s0959-8049(98)00030-6 9849481

[bib20] McCaffey J, Hilton S, Mazumdar M, Sadan S, Kelly WK, Scher HI, Bajorin DF (1997) Phase II trial of docetaxel in patients with advanced or metastatic transitional-cell carcinoma. J Clin Oncol 15: 1853–1857 9164195 10.1200/JCO.1997.15.5.1853

[bib21] Moore M, Tannock I, Ernst D, Huan S, Murray N (1997) Gemcitabine: a promising new agent in the treatment of advanced urothelial cancer. J Clin Oncol 15: 3441–3445 9396395 10.1200/JCO.1997.15.12.3441

[bib22] Moore MJ, Winquist E, Vokes EE, Hoving K, Stadler WM (2003) Phase II of oxaliplatin in patients with inoperable, locally advanced or metastatic transitional cell carcinoma of the urothelial tract who have received prior chemotherapy. Proc Am Soc Clin Oncol 22: 408, (A1638)

[bib23] Papamichael D, Gallagher CJ, Oliver RT, Johnson PW, Waxman J (1997) Phase II of study of paclitaxel in pretreated patients with locally advanced/metastatic cancer of the bladder and ureter. Br J Cancer 75: 606–607 9052419 10.1038/bjc.1997.106 PMC2063294

[bib24] Pronzato P, Vigani A, Pensa F, Vanoli M, Tano F, Vaira F (1997) Second line chemotherapy with ifosfamide as outpatient treatment for advanced bladder cancer. Am J Clin Oncol 20: 519–521 9345341 10.1097/00000421-199710000-00018

[bib25] Sridhar SS, Stadler W, Le L, Hedley D, Pond G, Wright J, Vokes E, Thomas S, Moore M (2005) Phase 2 study of bortezomib in advanced urothelial cancer. A trial of the Princess Margaret Hospital (PMH) Phase II Consortium. Proc Am Soc Clin Oncol 23(16S): 422s, (A4677)

[bib26] Stadler W, Kuzel T, Roth B, Raghavan D, Dorr FA (1997) Phase II study of single-agent gemcitabine in previously untreated patients with metastatic urothelial cancer. J Clin Oncol 15: 3394–3398 9363871 10.1200/JCO.1997.15.11.3394

[bib27] Sternberg CN, Yagoda A, Scher HI Watson RC, Herr HW, Morse MJ, Sogani PC, Vaughan Jr ED, Bander N, Weiselberg LR (1988) M-VAC (methotrexate, vinblastine, doxorubicin and cisplatin) for advanced transitional cell carcinoma of the urothelium. J Urol 139: 461–469 3343727 10.1016/s0022-5347(17)42494-3

[bib28] Sweeney C, Roth BJ, Kaufman DS, Nicol SJ; for the Hoosier Oncology Group, Indianapolis IN; Massachusetts General Hospital, Boston, MA; Eli Lilly and Company, Indianapolis, INA1653 (2003) Phase II study of pemetrexed for second-line treatment of transitional cell cancer of the bladder. Proc Am Soc Clin Oncol 22: 411, (A1653)

[bib29] SWOG, Green S, Weiss GR (1992) Southwest oncology group standard response criteria, endpoint definitions and toxicity criteria. Invest New Drugs 10: 239–253 1487397 10.1007/BF00944177

[bib30] Theodore C, Geoffrois L, Vermorken JB, Caponigro F, Fiedler W, Chollet P, Ravaud A, Peters GJ, De Balincourt C, Lacombe D, Fumoleau P (2005) Multicentre EORTC study 16997: feasibility and phase II trial of farnesyl transferase inhibitor & gemcitabine combination in salvage treatment of advanced urothelial tract cancers. Eur J Cancer 41(8): 1150–1157 15911238 10.1016/j.ejca.2005.02.015

[bib31] Vaughn D, Broome CM, Hussain M, Gutheil J, Markowitz A (2002) Phase II trial of weekly paclitaxel in patients with previously treated advanced urothelial cancer. J Clin Oncol 20: 937–940 11844814 10.1200/JCO.2002.20.4.937

[bib32] Von der Maase H, Hansen SW, Roberts JT, Dodliotti L, Oliver T, Moore MJ, Bodrogi P, Albers P, Knut A, Lippert CM, Kerbrat P, Sanchez Rovira P, Wersall P, Cleal SP, Roychowdhury DF, Tomlin I, Visseran-Grull CM, Conte PF (2000) Gemcitabine and cisplatin *vs* methotrexate, vinblastine, doxorubicin and cisplatin in advanced or metastatic bladder cancer: results of a large, randomised multinational, multicentre phase III study. J Clin Oncol 17: 3068–3077 10.1200/JCO.2000.18.17.3068 11001674

[bib33] WHO Handbook for Reporting Results of Cancer Treatment (1979). Geneva: World Health Organization

[bib34] Witte RS, Elson P, Bono B, Knop R, Richardson RR, Dreicer R, Loehrer PJ (1997) Eastern Cooperative Oncology Group Phase II trial of ifosfamide in the treatment of previously treated advanced urothelial carcinoma. J Clin Oncol 20: 589–593 10.1200/JCO.1997.15.2.589 9053481

[bib35] Wulfing C, Machiels J, Richel D, Grimm M, Treiber U, de Groot M, Beuzebec P, Farrell J, Stone NL, Leopold L, El-Hariry I (2005) A single arm, multicenter, open label, phase II study of lapatinib as 2L treatment of pts with locally advanced/metastatic transitional cell carcinoma of the urothelial tract. Proc Am Soc Clin Oncol 23(16S): 401s, (A4594)

